# The new contraceptive revolution: developing innovative products outside of industry^†,‡^

**DOI:** 10.1093/biolre/ioaa067

**Published:** 2020-05-09

**Authors:** Rebecca L Callahan, Neha J Mehta, Kavita Nanda, Gregory S Kopf

**Affiliations:** Reproductive Health Product Innovation & Introduction, Global Health Population & Nutrition, FHI 360, Durham, NC, USA

**Keywords:** pharmaceutical development, preclinical development, clinical trial, target product profile, first-in-human, user preferences, market research, acceptability, regulatory strategy

## Abstract

A significant global unmet need for new contraceptive options for both women and men remains due to side effect profiles, medical concerns, and inconvenience of many currently available products. The pharmaceutical industry has largely abandoned early research and development for contraception and will not likely engage to bring new products to the market unless they have been significantly de-risked by showing promise in early phase clinical trials. This lack of interest by big pharma comes at a time when scientific and technological advances in biology and medicine are creating more opportunities than ever for the development of new and innovative drug products. Novel partnerships between the academic sector, small biotechnology companies, foundations, non-government organizations (NGOs), and the federal government could accelerate the development of new contraceptive products. We discuss the challenges and opportunities that we have encountered as an NGO with a mission to develop novel contraceptive products for low- and middle-income countries and how it differs from developing products for higher-income markets. We hope that our experiences and “lessons learned” will be of value to others as they proceed down the product development path, be it for female or male or for hormonal or nonhormonal contraceptives.

## Teaser

Based on our experience and lessons learned as an NGO engaged in contraceptive research, we describe opportunities and challenges facing contraceptive developers outside the pharmaceutical industry across the stages of product development.

## Introduction

The introduction of the oral contraceptive pill in 1960 represents one of the most significant medical advances of the twentieth century [1]. “The pill,” along with subsequent forms of hormonal and nonhormonal (e.g., new barrier) contraception, gave women control over their own fertility and ignited a social revolution allowing women to plan their families and enter the work force. However, while important contraceptive advances have been made over the past six decades, the current method mix does not meet the range of women’s needs across their reproductive lifespans [2]. Women often discontinue use due to inconvenience, side effects (e.g., abnormal bleeding), and medical concerns (e.g., cardiovascular or cancer risk), which leads to unintended pregnancies and abortions. Many of these concerns relate to the contraceptive steroids that are used in these products. Moreover, with the exception of incremental improvements to condom design and vasectomy procedures, the contraceptive revolution has not included new methods for men. A clear need exists for innovative new products for both women and men that expand options, reduce side effects, and increase satisfaction and ease of use.

Given this obvious need for new and improved contraceptive technologies, why have research and development (R&D) efforts stalled? Multinational pharmaceutical companies (“big pharma,” e.g., Wyeth, Schering, Organon, Merck) abandoned both their female and male hormonal and nonhormonal drug discovery R&D programs in the early 2000s. Reasons include (1) the existence of many effective, low-cost products in the market, some over-the-counter; (2) the high bar for developing commercially successful new products with such highly effective existing options already marketed; (3) liability concerns since, unlike other therapeutics that treat a medical problem/disease, contraceptives are generally used for long periods of time by healthy individuals; and (4) the high cost of R&D. The arguments against investing in new male methods largely focused on the idea that women would not trust men to use them properly and that men do not get pregnant so would be less motivated to use. Similarly, companies that currently manufacture and/or market contraceptives are not motivated to develop new products since return on investment (ROI) with existing products is quite high (e.g., pills are inexpensive to manufacture).

This lack of interest by big pharma comes at a time when scientific and technological advances in biology, chemistry, medicine, engineering, and materials science are creating more opportunities than ever to develop novel products. Sequencing of and curating the human genome and the genomes of other animal species, as well as technological advances in both forward (e.g., GWAS) and reverse genetics (e.g., Crispr Cas 9) and the “omics” revolution, have provided more robust tools to identify causative factors in human disease, to identify and validate new pharmaceutical targets to treat disease, and to develop animal models that can mimic human disease or various medical conditions. Screening technologies have also advanced with the advent of DNA-encoded libraries and fragment-based drug discovery methodologies. Medicinal chemistry has benefitted from the use of artificial intelligence and machine learning. Pharmacogenetics and pharmacogenomics are providing greater insight to how the genome can influence drug response. Moreover, additional disciplines of engineering and materials science have intersected with biology and chemistry to provide new approaches to drug delivery.

While the above advances have been broadly applied to other areas of drug development, the exodus of big pharma from contraceptive R&D has certainly slowed the pace of the application of these novel scientific and technological approaches to contraception. Fortunately, however, the commitment of government, philanthropic organizations, and non-government organizations (NGOs) has kept the space alive. In fact, these non-commercial entities have played central roles in the development of most contraceptive products currently on the market. Basic research in reproduction supported largely by the US National Institute of Child Health and Development (NICHD) has formed the foundation of most contraceptive research. Preclinical and clinical development activities carried out by NGOs, such as the Population Council and FHI 360, and funded by federal agencies and philanthropic foundations have produced most of the long-acting, reversible contraceptives commercialized by the pharmaceutical industry (e.g., Paragard, Mirena, Jadelle, Norplant). Over the last 20 years, even in the absence of pharma support for contraceptive R&D, this non-pharma infrastructure has continued to foster the development of new and innovative contraceptive products, with scientists, clinicians, and entrepreneurs who are passionate about improving the contraceptive landscape. Importantly, however, any new product ultimately emerging from this non-pharma model will require the considerable financial, manufacturing, and commercialization capabilities of the pharmaceutical industry to reach the market. Thus, the building of viable academic–public–private partnerships is critical to success.

In this paper we describe our experience as a large, international NGO conducting contraceptive R&D outside of big pharma. For close to 50 years, FHI 360 has been developing and evaluating contraceptive technologies for use in low- and middle-income countries (LMICs). Our earliest efforts focused on field trial evaluations of intrauterine devices (IUDs), oral contraceptives, permanent contraception, and female barrier methods. Since then, our researchers have conducted hundreds of clinical trials to develop new contraceptive methods and assessed the safety, efficacy, and acceptability of most contraceptives marketed today. Our efforts have contributed to the development and/or introduction of more than 10 contraceptive products in more than 30 countries. Our team is composed of individuals with backgrounds in basic science, pharmaceutical development, clinical research, project management, epidemiology, and social/behavioral science. We work with big pharma, small biotech, and academic laboratories as our development partners to develop medium- to long-acting female contraceptives for women. Our funding largely comes from the Bill & Melinda Gates Foundation (BMGF) and the U.S. Agency for International Development (USAID), and we work collaboratively with personnel from both organizations. Our mission is to develop low-cost products that can be procured by agencies such as USAID for distribution in LMICs. With one exception, the products in our current development pipeline are hormonal and utilize well-characterized progestins (i.e., levonorgestrel, etonogestrel, medroxyprogesterone acetate). In addition to their low cost, the products in our pipeline are being developed to (1) be acceptable to women including offering potential for discreet use; (2) be stable under Zone 4b conditions, as cold-chain storage is not available in many LMIC settings; (3) deliver the lowest safe and effective steroid dose; and (4) be easily administered by lower-level healthcare workers or self-administered. These product characteristics, among others, impart a high bar on development and thus require innovative thinking and approaches.

**Figure 1:**
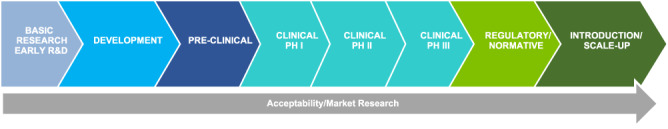
The stages of pharmaceutical product development.

We hope that our experiences, challenges, and “lessons learned” while carrying out our mission will be of value to others as they proceed down the contraceptive product development pathway (Figure 1).

## Discovery, development, and preclinical product development

Drug development is a complicated and highly integrated process that is both time- and finance-intensive. The following is a cursory overview of these processes using small molecules as an example (biologics represent a different process). The initial phase of basic research and early R&D is comprised of target identification and validation, screening for compounds against the target using in silico approaches and cell-based or non-cell-based assays with dynamic range outputs, design of compounds by computational chemistry, compound synthesis by organic chemists, and some initial medicinal chemistry to define active compound scaffolds. As projects proceed into development, medicinal chemistry efforts become more intensive with the goal of identifying lead compounds and backups. These compounds then move to preclinical pharmacology where both ADMET (absorption, distribution, metabolism, excretion, toxicity) and DMPK (drug metabolism and pharmacokinetics) activities are performed. An iterative cycle of medicinal chemistry activities further improves, if possible, undesirable compound characteristics identified in ADMET and DMPK studies. This iterative process may also include some in vivo efficacy testing. Later-stage compounds will also undergo additional toxicity testing (e.g., reproductive toxicity, genotoxicity, clastogenicity) and carcinogenicity testing under good laboratory practice (GLP) standards. Exploration of routes of chemical synthesis for lead compound scale-up often occurs in parallel with these activities. As compounds continue to advance in the drug development pipeline past the discovery stage and into the preclinical stage, chemistry, manufacturing, and controls (CMC) activities are initiated for proper analytical method identification and validation, and a regulatory strategy is developed. Scale-up to good manufacturing practice (GMP) for clinical trial batches is then initiated prior to Phase I safety, tolerance, and initial pharmacokinetic (PK) studies.

Recognizing that the success rate for bringing a new compound to market is extremely low, big pharma’s mantra is to “fail early and often,” and time is perceived as the biggest enemy for this long, complex, and expensive process. Industry institutes key milestones and go/no go criteria along the pathway to ensure objective and timely decision-making and to minimize non-productive activities. For industry, the focus is on the mission of the company, the goal of the therapeutic area, and ROI.

### Product development in academia

In contrast, while more academic institutions are building product development capacity, drug development in academic laboratories is less common and presents several challenges. Academic investigators are often not trained in the drug development process and, thus, may not appreciate how it differs from “pure” research for the pursuit and expansion of knowledge. Research is often the most important component in research-intensive universities, and an academic investigator’s currency for promotion is obtaining grants and publishing, along with teaching and service to the scientific community. Grants often make up a majority of an academic investigator’s funding, allowing pursuit of objectives to further the research literature, which may deviate from the initial aims of the grant. Although timelines and accomplishments are clearly important for grant renewal, grants are not usually as time and milestone driven as product development is in the pharmaceutical industry where adherence to clearly defined objectives and go/no go criteria is paramount. Therefore, since expectations for outcomes can be very different, funding of product development activities through a grant mechanism may not be optimal. For example, a grant proposal with great basic science objectives reviewed by product development experts might not fare well if the product development possibilities are not immediately obvious. Conversely, a solid product development strategy could be proposed, but reviewers from a basic research discipline might view the work as pedestrian and not scientifically interesting. Support to an academic investigator through a contract or cooperative agreement mechanism with defined, prioritized, and time-dependent deliverables can move work closer to the industry model and should be encouraged.

### Lessons learned from an NGO outsourcing model

This latter model is how we at FHI 360 collaborate for early-stage development. FHI 360 is an international NGO and not a university; we have limited laboratory-based facilities, so we use an outsourcing model engaging with multiple partners from academia, big pharma, small biotech, CROs, and other entities to carry out our mission. We first conduct due diligence on potential development partners who may have a novel drug delivery platform that could be used to develop a contraceptive satisfying the criteria outlined above. Through contracts with mutually agreed upon scopes of work, we provide both preclinical and clinical technical expertise as it relates to contraceptive development to our partners. We also utilize a cadre of consultants with expertise in various aspects of the drug development process (CMC, toxicology, regulatory, contract negotiation) and third-party vendors (API manufacturers/providers, animal PK, bioanalytical services, sterilization) for product development support, tailored to the particular needs of a development partner (Box 1).

Box 1. Recommendations for collaborating with academic product development partners

• Work proactively to clearly define goals and determine the scope of work.

• Define objective measures of progress and success early, and ensure everyone is in agreement (e.g., timelines, target product profiles, integrated product development plans).

• Ensure that the principal investigator, as well as the entire laboratory team, is invested in the project. Communication is key.

• Confirm alignment on project content and timelines for delivery of progress reports.

• Involve investigators when engaging with the technology transfer office around IP issues as they bring knowledge of the technology and background research.

We have learned several lessons from this model. First, as discussed above, working with academic institutions presents a unique set of challenges. Coming to agreement on contracts and budgets with university administration is time-consuming. Negotiating intellectual property rights can also take considerable effort as university technology transfer offices vary in their requirements, level of sophistication, and philosophy. Therefore, these negotiations must start early and be shepherded closely. Secondly, in terms of working with academic laboratories, we have learned that a team environment is critical to success, with involvement of both the principal investigator and other members of the laboratory analyzing results and working with us to set a path for future development adhering to our predetermined milestones and timelines. Also, because we have observed inconsistency in validated analytical procedures across laboratories—even core laboratories—we must closely review results to ensure quality and consistency across projects. Finally, we have learned that, though sometimes challenging given both our own and our partner’s scientific interests, keeping focus on objectivity is key. If agreed-upon milestones and specific development criteria cannot be met, the project must be terminated. As in industry, failing early is the most effective use of limited financial resources.

Although much of this discussion has focused on collaboration with academic investigators, we have experienced similar challenges with small companies (both established and startup). Often spun out of universities, start-up companies may have personnel with academic backgrounds, and, with a few employees, staff are often focused on multiple activities, leaving insufficient time for focus on project issues.

Finally, in addition to these partner-related lessons, we have also learned that early and thorough due diligence of third-party providers of product development services (e.g., bioanalytical, toxicology, sterilization) is critical. Centralization of these services to ensure that all development partners utilize the same service providers is also the most time- and cost-efficient approach, avoiding the need for duplicating activities such as validating analytical test methods.

## Regulatory considerations

### Setting goals: defining the product and the pathway

A key product development tool that we use in our contraceptive development programs is the target product profile (TPP). As with any other pharmaceutical, the development of a new contraceptive requires that we have a goal for the final product attributes. The FDA has detailed draft guidance on the development of TPPs (Target Product Profile) [3], which can be viewed as a summary of the eventual product label and product goals. Working backwards from the imagined final product can help to shape and inform the product development program. While TPPs can and should evolve during the development process, developing an initial TPP can provide an early focus for a development program. Ideally, contraceptive developers should create a basic TPP early in product development; refer to it regularly while designing the preclinical, clinical, and regulatory strategy; and update it as needed. A well-developed TPP helps to align objectives across different functional areas (preclinical, CMC, clinical) and can accelerate timelines, minimize risks, and eventually lead to an optimal product.

In addition to a TPP, new products should also be guided by a strategic development plan created early in the development process. Such a plan may include CMC, regulatory, and clinical development activities. For example, the regulatory component of the strategic development plan is critical for guiding both regulatory approval steps and life cycle management of the product. Designing effective product development plans includes knowing the target label (as defined by your TPP) and the target market. As described above, defining metrics and criteria for success, e.g., go/no go decisions, and allowing for “early kill” are important components of the plan.

### Regulatory overview

The International Council for Harmonisation (ICH) is the association that harmonizes guidelines for global pharmaceutical development as well as their regulation. Its members are stringent regulatory agencies (SRAs), which include the US Food and Drug Administration (FDA) and the European Medicines Agency (EMA). The US FDA is a single, centralized drug regulatory agency, while the European Union (EU) has both centralized (EMA) and decentralized agencies throughout its member states. Even so, the clinical regulatory drug development processes between the US and in Europe are very similar. Both require permission to conduct clinical trials based on established preclinical safety of the test product (see *Animal studies)*, and both follow similar phases of clinical studies for product development. Generally, Phase 0 or 1 studies are first conducted in a small number of healthy participants to clarify pharmacology or dose, followed by larger Phase 2 studies to determine dose/response relationship and lastly by larger Phase 3 trials to demonstrate efficacy. Safety is assessed across all phases. In the United States, obtaining final drug approval requires a new drug application (NDA) to the FDA, whereas in the EU there are four pathways to approval: (1) centralized through the EMA, (2) application to the regulatory body in a single EU state, (3) mutual recognition (after approval in a single state, recognition in all states via the EMA), and (4) a decentralized process (simultaneous application in multiple EU states)*.*

While approval pathways are generally similar across regulatory agencies, we have learned that viewpoints may vary substantially. One example is the difference in classification of copper T IUDs, which the EU defines as devices and the FDA considers as a drug-device combination product, resulting in substantially different regulatory requirements for approval.

### Choosing a regulatory pathway

As is true for the pharmaceutical industry, determination of the appropriate regulatory pathway for our drug development programs is often driven by marketing goals. Demand forecasting allows us and/or potential investment partners to determine which countries to consider for regulatory approval of a new contraceptive product. FHI 360’s mission and that of our funders, informed by our acceptability research, generally guide the selection of intended LMIC countries for product introduction and delivery. While the pharmaceutical industry will invariably target SRAs in the United States or Europe for their initial marketing authorizations, our group has considered more innovative, non-SRA, regulatory pathways to speed access to new and/or affordable contraceptives in LMICs. Although “regulatory innovation” at a procedural level has proven to be challenging, in part because of the role of regulatory agencies to focus on ensuring sufficient safety data for new products, we try and incorporate innovative regulatory strategies within our development programs. For example, we recently proposed and received agreement from EU regulators on the use of pharmacokinetic (PK) modeling and simulation to support a reduced size and scope of a pivotal efficacy trial. We continue to monitor and learn about the regulatory feasibility, requirements, cost, and timelines of current alternative procedures meant to streamline regulatory reviews and approvals. Currently, our continued reliance on SRA approval for product registration in many LMICs, in addition to the need to attract potential commercialization partners to progress our development programs, has led us to primarily target SRA approval for most of our products. A single SRA approval is sufficient for the introduction of products into the global distribution chain in most LMICs by procurement agencies such as USAID and the United Nations Population Fund (UNFPA).

### Regulatory guidance for contraceptive development

In 2005, the EMA issued a guideline for clinical investigations of steroid contraceptives to help developers in various aspects of study design [4]. Our team has used this as key guidance to inform our contraceptive development programs. In 2019, the FDA released draft guidance for public comment on establishing safety and efficacy for hormonal contraceptives, which is largely aligned with the EMA guidelines [5]. While these documents are incredibly helpful, important gaps remain in terms of regulatory guidance for contraceptive development, namely, for nonhormonal female methods and all forms of male contraception. The relative lack of contraceptive product development activities for men has resulted in very little guidance from regulators for these programs. One of the only FDA guidances that was previously available to developers, and was subsequently withdrawn, was focused on considerations related to the potential for adverse effects on the conceptus of a sexual partner who is or may become pregnant in clinical trials of non-contraceptive products involving male subjects [FDA draft guidance, Assessment of Male-Mediated Developmental Risk for Pharmaceuticals (2015)]. Given the lack of regulatory guidance for male contraceptives, as well as for nonhormonal female products, early engagement with regulatory agencies is strongly encouraged to jointly agree on a regulatory path forward. Working in close collaboration with regulatory agencies to develop a regulatory path forward for these new and innovative products, for which there is no precedence, could be a great opportunity for investigators in this field to shape this critical component of the product development pathway (Box 2).

Box 2. Regulatory lessons learned

• Identify key markets for your product early in development to target the appropriate regulatory pathway.

• Evaluate product development programs of similar approved products to inform your own.

• Refer to available contraceptive development regulatory guidances to design your development program.

• Consider innovation within your product development plan to reduce time and cost to approval.

• Engage regulatory authorities early and often to ensure alignment on your product development plan.

### Engagement with regulatory authorities

An important lesson we have learned is that regulatory preparation for new product development takes time. Engaging with the FDA (or other SRA), preparing submission packages, meeting with the regulators and responding to feedback to begin a first-in-human (FIH) study is a lengthy and complicated process. Additionally, substantial time is required to adequately prepare for, get ethics approval for, and document the FIH study and provide the necessary (and intense) training for investigators and study staff. In addition to being time-consuming, these activities require substantial resources and funding.

### Animal studies

Choosing the right animal studies is key to producing the appropriate data package on which to base key clinical trial decisions for the rest of the product development program. First, regarding toxicity, the FDA has recommendations regarding the preclinical studies needed, but a full package of toxicity studies is not always required for initial human studies. When using an active pharmaceutical ingredient (API) already in approved products (i.e., not a new chemical entity (NCE)), as is the case with most contraceptive hormones, a shorter regulatory approval route ([505(b)(2)] NDA) can be followed, which relies on published safety data from an approved listed drug. However, careful strategic planning is required to determine what can be relied on from approved products and what additional toxicity testing is necessary; sometimes negotiations with the FDA or other regulatory agency around these points may delay study start. Second, regarding PK, it is critical to ensure that nonclinical studies use the right animal model, right doses, and right duration to allow informed dose selection for the first clinical trial.

## Clinical development

### Clinical trial considerations

Clinical trials for contraceptive products present a unique set of considerations given the population (young, healthy individuals) and outcome (pregnancy) of interest. Selecting the right inclusion criteria at all stages of clinical development to maximize the collection of necessary and appropriate data yet not limit/inhibit enrollment can be challenging. For example, for Phase 1 trials of female methods, requiring women to be at low risk of pregnancy yet not using hormonal contraceptives can lead to difficulties in recruitment. For early phase male contraceptive studies, defining potential risk to a female partner must also be considered (e.g., if a drug would be found in semen to which women would be exposed). Selecting the right trial sites is also important. For example, academic centers, while frequently having experienced investigators, might not be the best settings for Phase 1 trials due to slower enrollment. If rapid, efficient recruitment and extensive PK sampling and frequent visits are needed, dedicated Phase 1 facilities might be a better option. With regard to contraceptive steroid analysis, choosing the right laboratory and assays for PK analyses is important. For trials of male contraceptives, ensuring alignment and quality control of laboratories doing semen evaluations would be important.

In the first few small studies of methods with NCEs, adverse event (AE) and serious adverse event (SAE) numbers are likely to be very low and possibly unrepresentative of the safety profile of the investigational product. This can make signal detection difficult. Signal detection in pharmacovigilance involves looking at cumulative adverse reaction data for patterns that suggest new safety concerns. One potential way to improve on signal detection is to collaborate with other investigators using the same API and create platforms for sharing of safety data.

### The role of pharmacokinetics

For many drugs in other areas, the therapeutic window, or the range of drug doses which are highly effective while still safe, can be readily characterized. This, however, is not as straightforward for contraceptive steroids. While initial clinical trials define the PK of a new formulation, the reliance on PK to determine appropriate doses for further efficacy evaluation can be complicated. Even for methods such as contraceptive implants which release fairly constant levels of drug over many years, the contraceptive threshold in terms of blood levels that maintain a high level of effectiveness remains elusive and may be influenced by multiple factors [6]. The presumed contraceptive threshold for levonorgestrel implants, which do not consistently suppress ovulation, was determined by retrospectively evaluating serum hormone levels above which pregnancies were consistently prevented. In contrast, for etonogestrel implants, the presumed contraceptive threshold was based on a level at which most women had suppression of ovulation (a surrogate marker of effectiveness in pregnancy prevention). While surrogate markers such as ovulation inhibition are more likely to indicate risk of pregnancy than PK alone, they are not necessarily completely predictive. For example, even if ovulation occurs, it may be abnormal, or other effects of hormonal contraceptives, such as thickening of cervical mucus and thinning of the uterine endometrium, may prevent conception and implantation even if ovulation occurs. These issues should also be considered for any new nonhormonal female or male contraceptive.

Pharmacokinetic parameters of contraceptive steroids also vary greatly within and between individuals. Part of this variability may be due to assay variations (i.e., in historical published literature) as well as true ethnic differences in the amount of hepatic metabolism. Factors such as diet, concurrent illness, smoking, or weight may also affect steroid PK, further complicating study population selection. These factors must be considered when selecting clinical sites and developing study eligibility criteria. The selection of study populations may limit labeling and should be considered in product development planning. While our experience is primarily with contraceptive steroids, PK variability is seen with many other drugs as well.

### Drug–drug interactions

Early in development, developers should also consider how the effectiveness of a drug might be influenced by other drugs or other things that could influence metabolism of the drug (e.g., grapefruit juice). The FDA has recently put out guidance on the design of drug–drug interaction (DDI) studies to help investigators design and evaluate DDIs studies during development [7]. Individuals often use more than one drug at a time, and this could compromise the effectiveness or safety of either drug and lead to morbidity and mortality. Not knowing about other drug use can also compromise data and study conclusions (e.g., PK and/or safety assessments). DDIs are particularly relevant for hormonal contraceptive methods. In the liver, cytochrome P450 enzymes catalyze the most important metabolic reactions, with the most significant for progestin metabolism being cytochrome P450 3A, particularly polypeptide 4 (CYP3A4). Many progestins are substrates for CYP3A4, and different progestins themselves have varying effects on CYP enzymes. The effectiveness of any contraceptive may be compromised if PK parameters are affected by other drugs to such an extent that primary mechanisms of action are inhibited. Contraceptives may also affect the metabolism of other drugs, leading to issues with effectiveness or safety of the co-administered medication, and in vitro assays may not predict in vivo drug interactions. Further, these interactions may be complex, particularly when multiple, possibly interacting, drugs are involved and used long term. For progestin-only contraceptives, DDIs with liver enzyme-inducing medications such as antiretrovirals have led to decreases in effectiveness of even highly effective long-acting contraceptive methods, such as implants [8]. In addition, there may be polymorphisms of CYP3A4 genes in various populations, leading to differences in metabolism of contraceptive steroids [9] and other drugs metabolized by this enzyme.

### Unique considerations for male methods

Finally, unique new challenges may occur with newer nonhormonal methods. In women, suppression of ovulation is achieved for currently available methods by suppression of gonadotropins. In men, suppression of sperm production may occur at various levels, all with their own unique challenges and potential adverse effects. New ethical issues may also arise. For example, newer methods in development may modify the germline. In such cases concerns are increased regarding the impact of unintended pregnancy, particularly on male fetuses.

### Additional lessons learned from clinical trials for female contraceptives

FHI 360 has been leading contraceptive clinical trials for decades. Again, due in large part to the lack of pharmaceutical industry interest in developing new contraceptive products, particularly for use in LMICs, international organizations like FHI 360 have led clinical development programs of many technologies. Although most of our previous work has been in LMICs, much of the experience we have gained is also relevant to high-income countries (HICs).

One ubiquitous lesson we have learned in this long history is that consistently high effectiveness in preventing pregnancy requires longer-acting methods (generally preventing pregnancy for at least a month). With short-acting methods (e.g., pills, barriers), effectiveness differs between perfect use and typical use [10]. Perfect use effectiveness rates are calculated using consistent and correct use, while typical use effectiveness generally refers to effectiveness while a method is reportedly being used but may not be used consistently, continuously, or correctly. Inconsistent or incorrect use leads to unintended pregnancy in many users [11]. Long-acting methods that do not require user action, such as IUDs and implants, lead to higher effectiveness, with both very high typical and perfect use effectiveness rates [12]. Partly because of this potential for high effectiveness, as well as user preference, which we have discovered through acceptability research described in the next section, our recent development work has focused on longer-acting methods such as injectables, IUDs, and implants (Box 3).

Box 3. Lessons learned from female contraceptive development

• High effectiveness requires long-acting forgettable methods, such as implants or longer-acting injectables.

• Self-injection improves adherence to injectable contraceptives.

• Some delivery systems such as implants require training and support to ensure that insertion and removal challenges do not occur when used at scale.

• Biodegradable implants have the potential to mitigate removal challenges and reduce burdens on the healthcare system by eliminating the need for removal.

Injectable contraceptives remain very popular among users, particularly in low-resource settings. However, intramuscular injectables such as DMPA-IM (e.g., Depo-Provera), which are intermediate acting but require some user action (reinjection every 3 months) also have lower typical use effectiveness than perfect use effectiveness due to failure to get reinjections on time. Although method-related concerns, such as side effects, are the most commonly reported reasons for discontinuation of injectables, access to reinjection services also remains a problem in low-resource settings [13]. Our research has shown that even a 3-month interval between injections is ultimately too frequent for many women, spurring interest in the development of a longer-acting product. Less frequent clinic visits would also reduce burden on women and providers [13–15].

In many LMIC settings, access to healthcare providers is limited. Even in HICs, discontinuation of injectable contraceptives due to failure to return for reinjection (due to inconvenience or cost) may be an issue. Thus, other efforts to improve method continuation rates have evaluated self-care. The WHO defines self-care as “the ability of individuals, families and communities to promote and maintain health, prevent disease, and cope with illness and disability with or without the support of a healthcare provider” [16]. Part of our research efforts thus focus on new longer-acting contraceptive technologies that have the potential for self-administration. Our research has also shown that self-administration of currently available contraceptives can improve method continuation rates. In a randomized trial of an existing subcutaneous 3-month DMPA formulation (Sayana Press), we found that a significantly higher rate of 1-year continuation among women who self-injected compared with those assigned to return to a provider for the injections [17]. Self-administration also has the potential to improve method continuation in the United States and other HICs [18].

While long-acting methods such as IUDs and contraceptive implants have led to improved effectiveness comparable to permanent contraception, the insertion and removal of long-acting, provider-administered methods can be challenging. Insertion and removal issues are often not evident in the highly structured and controlled clinical trials used for method approval or even in initial introductory trials. When used at larger scale, however, especially in lower-resource settings, methods requiring insertion by a trained provider may have lower effectiveness due to variations in successful administration. Additionally, removal issues may also present problems [19, 20]. Thus, introduction of methods such as implants should be accompanied by extensive training on insertion and removal as well as method-related counseling. To mitigate issues with removal of contraceptive implants, we are investigating the use of biodegradable implants which, though requiring a provider for insertion, will not require removal, thus reducing this burden on the healthcare system.

## Product acceptability and market research

### Understanding the global contraceptive market

Like any drug or device, proven safety and efficacy are required for a new contraceptive to reach the market. However, unlike most other pharmaceuticals, contraceptives are used by young, healthy people to prevent a condition (pregnancy) rather than treat an ailment. Therefore, not only are the safety and efficacy bars for contraceptives among the highest of any drug class, but consumers also have very high expectations for these products. For many, side effects and the non-contraceptive “side benefits” are as important—if not more so—than contraceptive efficacy in users’ choice between different products. Contraceptives are ultimately consumer products, and just like any consumer product, market research is key for developing something that people want to use.

Historically, however, contraceptive development efforts, even in industry, have focused little attention on what women (and men) want in their method, leaving technical feasibility to dictate design decisions. Given that the majority of women cite product-related side effects and health concerns as the reason for stopping use [21], in our programs we have prioritized better understanding of what users will accept and desire in their contraceptives in order to develop products that users will (hopefully) adopt, use correctly, and continue.

Globally, more than half of women have ever used a modern method of family planning, and contraceptive prevalence is only increasing [22]. In the United States, more than 99% of women who have ever had sex report having used a method at least once [23]. And, of course, women are not the only users of contraception—globally, male method use accounts for 25% of contraceptive prevalence (with substantial geographic variation) [22]. Though use is high, as mentioned earlier, so is discontinuation and product dissatisfaction. An individual’s perceptions of and preferences for contraceptive characteristics are influenced by factors at multiple levels from the familial and community to the broader social and cultural. Where an individual is in their reproductive life cycle, their level of self-efficacy and personal agency to make decisions about their own health and contraceptive use and cultural norms and beliefs associated with menstruation and reproduction all influence contraceptive preference and method acceptability. So how do we determine what users want given the size, variation, and multifaceted nature of the contraceptive market?

### How and when to collect user preference data

An important first step is to define key user groups, or market segments, in different geographies. While not the same as a market analysis to determine financial potential of a new product, this assessment is important for gauging the potential success of a new product. Most public sector and foundation-funded contraceptive development efforts are focused on low-resource populations, often in LMICs. For affordable products to be made available in these settings, however, developed country markets must be established to subsidize affordable access. Understanding what product characteristics are more or less important in low- and high-income countries is, therefore, required. Preferences will vary in both settings based on a myriad of factors including prior contraceptive experience, what methods are readily available in an individual’s context (familiarity), future childbearing intentions, preferences for hormonal versus nonhormonal methods, influence from partner, family, and friends, etc.

A more nuanced understanding of user needs may be gleaned through the development of user profiles in different target geographies. Beginning with categories of users (e.g., adolescents; those interested in spacing their next pregnancy versus limiting childbearing; rural versus urban dwellers), more distinctive and useful profiles can be generated through participatory data collection techniques, revealing more specific needs and determinants of those needs. A recent example of this kind of user profile generation was part of the Contraceptive Technology (CT) Innovation Lab effort that utilized a human-centered design approach to generate new contraceptive technology ideas in India and Kenya [24]. Some examples of profiles uncovered across the reproductive life course included the “Determined Dreamer” and “Regretful Teen Mom” in adolescence and the “Stability Seeker” and “Contented Homemaker” later in life. Profiles such as these can highlight unique contraceptive needs and preferences for product designs that often resonate across populations [24].

User preference data can and should be collected at all stages of product development. In order to inform early design decision-making and R&D investment, potential end-users (and providers) can be presented with a product concept and asked their views including whether they would be interested in using it if it were available. Both qualitative, e.g., focus group discussions (FGDs), and in-depth interviews or quantitative survey questions can be used to collect these data. Mixed method approaches can be particularly effective at this early stage. For example, qualitative interviews can be used to help define a set of product characteristics to be included in a quantitative discrete choice experiment (DCE) survey, which is a useful method for measuring the relative importance of specific product characteristics. DCEs have been used to inform the design of sexual and reproductive health outreach services for youth in Malawi [25], preferences for place of delivery in rural Tanzania [26], and HIV prevention methods among high-risk populations in South Africa [27], among many other examples. Additionally, as mentioned above, user-centered design techniques including rapid, participatory data collection and ideation workshops are increasingly being employed to generate novel, “out of the box” ideas for new products.

While the benefits of collecting user input at the earliest stages or even prior to the start of product development are obvious, the limitations of these data should also be considered. Hypothetical questions about product use are not necessarily reliable—what people say they will do or use often does not predict actual behavior [28]. Go/no go development decisions should not be made solely on hypothetical input; however, these kinds of data can help identify major gaps, problems, and design opportunities to create desirable products (Box 4).

Box 4. Acceptability and market research considerations

• Contraceptives are consumer products, and user preference research is key to developing methods that people want to use.

• Unlike most other pharmaceuticals, contraceptives are used by young, healthy people who not only expect few product side effects but often desire “side benefits” from their contraceptive method.

• User preference research should be conducted throughout the product development process using appropriate methods for the stage of development.

• User-centered design approaches can be employed to develop user profiles and involve potential users in the design of products that best meet their unique needs.

• Creative strategies can be employed to solicit important feedback from clinical trial participants not necessarily related to safety and effectiveness but still crucial for developing a successful product.

More reliable user input can be solicited at the prototype or FIH stage of development (for some products) when potential users can actually try the product or product simulation. At this stage, perceptibility studies can be used to solicit user feedback on the feel and experience of a new technology. Perceptibility studies have been used in the HIV prevention field to explore user experience with vaginal prevention modalities including gels, tablets, and films [29, 30]. These studies are important for identifying product characteristics particularly related to sensory perceptions and formulation experience, e.g., perceptions of wetness, messiness, and leakage. Similarly, designing usability studies with early product prototypes is important for identifying potential use challenges before a product enters later, more expensive clinical testing.

Once a product does enter the latter phases of clinical testing, however, user feedback should continue to be collected. While clinical trials represent a unique context and participant experience is often quite different than “real world” conditions, they still offer an opportunity to collect actual user and provider experience with a new technology. Often the user acceptability component of efficacy studies includes a limited set of questions focused on user satisfaction and the potential for future use of the product. More creative acceptability components could be considered including asking trial participants about any benefits or challenges they experienced using the product and about how they perceived the counseling they received, i.e., was it beneficial? Could it be improved? Participants could be asked about their views on particular side effects and how they may have impacted their lives. While not all side effects are clinically relevant to safety or efficacy, some may have important implications for user acceptability and satisfaction [31].

## Manufacturing and commercialization

### Focus on business development

As this review illustrates, product development is a long process with multiple components and decision points. Especially early in the process, attention is focused on preclinical work and meeting the necessary requirements to move to later stages of development, often leaving the identification of a clinical manufacturing and commercialization partner to fall to the bottom of the priority list. Yet, in our experience, initiation of the “business development” side of the product life cycle cannot come too soon and is a discipline that requires a unique understanding of product development, sales, and negotiation. As scientists, we generally lack the necessary skills and experience required for the successful development of product marketing strategies and need strong business development support to realize our products’ potential. The industry recognizes this critical component and generally has full-time business development personnel embedded across therapeutic areas. In academia, such business development activity generally resides within the technology transfer office which, while understandable, is not optimal since these offices service multiple disciplines and therapeutic areas. For our program at FHI 360, we utilize business development consultants since we do not have this expertise in house.

As we move towards FIH clinical trials with our products, decisions first need to be made regarding Phase I and II manufacturing. Generally, product for these early phase trials is not made on a commercial scale and can be outsourced to a third-party clinical manufacturing organization (CMO) as a fee for service. Our team, led by our Director of CMC, negotiates this process. Since Phase I and II results often lead to further formulation refinement, going to a full-scale commercial process at this early stage is not warranted (Box 5).

Box 5. Marketing and commercialization recommendations

• Engage potential manufacturing/commercialization partners early in the development process. Establish a relationship and keep them updated on progress. Provide a brief, nonconfidential summary of your technology.

• Engage business development expertise early in the process to develop a strategic business plan.

• Involve your institution in business plan strategy development and potential deal scenarios, and think through backup alternatives.

• Present your expectations transparently to any potential manufacturing/commercialization partners (e.g., if your product is to be procured by agencies for LMICs and tiered pricing will be necessary).

• Maintain realistic expectations.

• Approach every deal as a unique process; no two deals are ever the same!

Identification of and initial negotiation with a potential manufacturer/commercialization partner should occur in parallel with these early clinical activities. We face a unique challenge in these efforts given the mission of our group, which is in part dictated by our funders (primarily BMGF and USAID) to develop and provide low-cost products for LMICs. Procurement agencies such as USAID must be able to purchase large quantities of the eventual products for distribution to developing markets. The challenge to any potential commercial partner, therefore, is to make a large enough profit margin in HIC markets and/or private markets in LMICs to offset a low LMIC procurement price. To address this, we have developed an innovative product portfolio to meet the needs of users around the world. However, the requirement for a tiered pricing system adds complexity to the negotiation with potential manufacturing/commercialization partners, as it requires market analysis in HICs as well as an understanding of potential product demand in LMICs. While our efforts to better understand user preferences and acceptability described in the last section help with this, the need for tiered pricing certainly affects our ability to negotiate revenue sharing terms.

### Potential partners

Several types of companies can be engaged when moving towards the manufacturing/commercialization stage. Smaller women’s health specialty companies are often looking for later-stage products to fill their development pipeline and satisfy investors. A challenge with these companies is that they often need to raise capital to successfully enter and consummate a deal. While larger branded and generic pharmaceutical companies are also interested in later-stage projects (generally Phase II and later), they generally have greater internal financial and human capital to bring to the table. For branded companies, lack of interest in early stage products is linked to their abandonment of the contraceptive R&D space as described earlier, whereas for generic companies, a lack of early R&D capability dictates a focus on later-stage leads. The common denominator for all companies looking to bring a new product into their portfolio is the ability to realize ROI in HICs without cannibalizing the market share of products in their current portfolio. A tiered pricing system to provide commodities at vastly lower prices for procurement agencies plays a significant role in what any deal might ultimately look like.

## Conclusion

The contraceptive product development landscape is ripe with opportunity given the tremendous advances in biology, chemistry, medicine, and engineering that could be applied to contraception for both women and men. While big pharma has largely abandoned this space, contraceptive development activities have been buoyed among NGOs, academia, and small business by renewed public sector and philanthropic commitment. Though these smaller actors face a steep learning curve to develop safe, effective, and appealing products while navigating a change and, in some instance, non-existent regulatory environment (e.g., nonhormonal and male methods), the substantial global need for new and improved contraceptive products that better meet users’ needs offers both a public health and market imperative for continued focus. We hope that our experiences and lessons learned blazing this new trail in contraceptive development outside the traditional pharmaceutical industry model will be beneficial for others engaging in this exciting and rewarding field. We are optimistic that new products coming from this consortium of non-traditional development partners will ultimately be of interest to big pharma, resulting in a wider range of contraceptives coming to market to meet the needs of all women and men.
